# Caloric Restriction and Telomere Preservation in *TERT* Knockout Adipocyte Progenitors Does Not Rescue Mice From Metabolic Dysfunction due to a *TERT* Function in Adipocyte Mitochondria

**DOI:** 10.1111/acel.14499

**Published:** 2025-02-11

**Authors:** Zhanguo Gao, Yongmei Yu, Kristin Eckel‐Mahan, Mikhail G. Kolonin

**Affiliations:** ^1^ The Brown Foundation Institute of Molecular Medicine University of Texas Health Science Center Houston Texas USA

**Keywords:** adipocyte, mitochondria, progenitor, senescence, telomerase, telomere, TERT

## Abstract

Inactivation of telomerase (TERT) in adipocyte progenitor cells (APC) expedites telomere attrition, and the onset of diabetes in mice fed high‐fat diet (HFD), which promotes APC over‐proliferation and replicative senescence. Here, we show that time‐restricted feeding or caloric restriction in the postnatal development of mice subsequently subjected to HFD prevents telomere attrition but not glucose intolerance. This metabolic effect of dietary intervention was not observed for mice with TERT KO in endothelial or myeloid cells. To characterize the telomere‐independent effects of *TERT* in the APC lineage, we analyzed mice with *TERT* knockout in mature adipocytes (AD‐TERT‐KO), which do not proliferate and avoid telomere attrition. Analysis of adipocytes from AD‐TERT‐KO mice indicated reliance on glycolysis and decreased mitochondrial oxidative metabolism. We show that AD‐TERT‐KO mice have reduced cold tolerance and metabolism abnormality indicating a defect in adaptive thermogenesis, characteristic of aging. Conversely, ectopic TERT expression in brown adipocytes‐induced mitochondrial oxidation and thermogenic gene expression. We conclude that TERT plays an important non‐canonical function in the mitochondria of adipocytes.

## Graphical abstract

This study demonstrates that TERT in mesenchymal cells is important not only for telomere maintenance but also for mitochondrial function. This was revealed in mice lacking TERT in mesenchymal progenitors and retained telomeres due to calorie restriction and in mice lacking TERT in adipocytes, which both displayed abnormal glucose metabolism. TERT was found to play an important role in mitochondrial biogenesis and oxidation in brown adipocytes and their adaptive thermogenesis function in male mice.

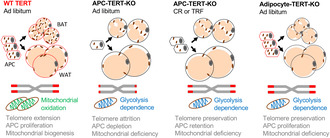

## 1 Introduction

Adipose tissue (AT), which overgrows in obesity, serves as an endocrine organ that regulates energy balance and metabolism. Expansion of white adipose tissue (WAT) by increased triglyceride storage in adipocytes (Friedman 2009) becomes pathogenic once this capacity is exceeded, resulting in lipid deposition in other organs (Johnson and Olefsky 2013; Powell‐Wiley et al. 2021). While the lipid‐storing adipocytes make up the majority of AT volume, its other cells including adipocyte progenitor cells (APC), endothelial cells (EC), and immune cells, with macrophages being most abundant, play important roles. AT health depends on the ability of APC to replace dysfunctional adipocytes (Eckel‐Mahan, Ribas Latre, and Kolonin 2020). Exhaustion of the APC pool, or of its proliferative potential, results in adipocyte hypertrophy, leading to their increased death, AT inflammation, fibrosis, dyslipidemia, and lipotoxicity setting the stage for insulin resistance and type‐2 diabetes development (Ghaben and Scherer 2019). Metabolic disease typically ensues due to hypoxia and death of hypertrophic adipocytes in visceral adipose tissue (VAT) (Roden and Shulman 2019). In contrast to VAT, subcutaneous adipose tissue (SAT) can play an anti‐obesity role (Porter et al. 2009; Tran and Kahn 2010). In response to the β‐adrenergic signaling, SAT adipocytes are induced to undergo brown adipogenesis (Bartness et al. 2014; Ducharme and Bickel 2008; Granneman and Moore 2008), relying on mitochondrial biogenesis (Nedergaard and Cannon 2014). The organ specialized in this function is brown adipose tissue (BAT) (Cinti 2009; Koza et al. 2000; Seale et al. 2008), which enables non‐shivering thermogenesis (Dulloo and Miller 1984). Inducible brown‐like (beige) adipocytes, activatable mainly in SAT, are functionally similar to BAT adipocytes (Kajimura, Spiegelman, and Seale 2015; Orci et al. 2004; Wu et al. 2012). Adaptive thermogenesis is enabled by uncoupling protein 1 (UCP1), and other mechanisms, that uncouple mitochondrial electron transport from ATP synthesis, resulting in heat dissipation (Collins, Yehuda‐Shnaidman, and Wang 2010; Nedergaard and Cannon 2014). By increasing energy expenditure, BAT and beige AT can normalize glucose homeostasis (Almind et al. 2007; Cohen et al. 2014; Lowell et al. 1993; Tseng, Cypess, and Kahn 2010).

Recent research on APC proliferation has highlighted its significance in maintaining healthy AT in the context of overnutrition and aging (Lin et al. 2024). Physiological changes accompanying aging result from cells in AT and other organs undergoing senescence (Borghesan et al. 2020; Fossel et al. 2022). Cell senescence is the state of proliferation arrest linked with the senescence‐associated secretory phenotype and increased expression of senescence‐associated beta‐galactosidase (SA‐βgal) and DNA damage response genes (Liu et al. 2019; Ogrodnik et al. 2024; Zhang et al. 2022). Accumulation of senescent cells contributes to changes leading to metabolic dysfunction (Borghesan et al. 2020; Gorgoulis et al. 2019). Aging‐related and overfeeding‐induced senescence in APC has been reported and shown to compromise the thermogenic capacity of brown adipocytes (Berry et al. 2017; Palmer et al. 2019; Schafer et al. 2016; Smith et al. 2021). Cell senescence typically arises due to critical telomere shortening and/or DNA damage elsewhere in the chromosomes or mitochondria (Baker, Narita, and Munoz‐Canoves 2023; Bloom et al. 2022; Han and Kim 2023; Wiley et al. 2016; Zhang et al. 2022). Telomerase (TERT), an enzyme‐extending telomeres, is fundamental for sustained cell proliferation capacity and prevention of cell senescence (Chakravarti, LaBella, and DePinho 2021). In humans, TERT is active in stem cells but is turned off in early development, which opens the path to telomere erosion and cell aging. While humans are born with telomeres in a 10–15 kb range, C57BL/6 mice used in research have telomeres over 50 kb and continue to express TERT in somatic cells, which makes mice suboptimal as a model of replicative senescence (Kipling and Cooke 1990; Prowse and Greider 1995). To overcome this limitation and study APC senescence in mice, we have created mouse *Tert* knockout models based on the notion that APC express platelet‐derived growth factor receptors (PDGFRs) (Gao et al. 2018; Traktuev et al. 2008). We have reported that mice lacking TERT in cells of *Pdgfrb* + lineage (APC‐TERT‐KO) undergo premature APC senescence linked with increased insulin resistance and glucose intolerance (Gao et al. 2020).

Approaches to suppress or reverse senescence would be integral in preventing and treating aging‐associated diseases (Fossel et al. 2022). It has been reported that in mice high‐calorie/high‐fat diet (HFD) feeding disrupts the circadian rhythm of APC division and leads to their continuous proliferation (Ribas‐Latre et al. 2021). Consistent with the expectation of uncontrolled proliferation to exhaust the APC pool without telomere maintenance, HFD expedited telomere attrition and aggravated metabolic disease in mice with APC *Tert* KO (Gao et al. 2020). It has been reported that time‐restricted feeding (TRF) is beneficial in the context of diet‐induced obesity and has been shown to improve glucose metabolism (Chaix et al. 2019; Hatori et al. 2012). This could in part be explained by the maintenance of functional APC through limiting their proliferation and telomere attrition. Here, to test this hypothesis, we investigated if telomere attrition can be delayed in APC‐TERT‐KO mice by dietary interventions in early postnatal development. We show that TRF prevents premature telomere attrition in APC‐TERT‐KO mice subsequently fed HFD. Post‐natal 30% caloric restriction (CR) also prevented telomere attrition in mice subsequently subjected to HFD feeding. However, post‐CR, APC‐TERT‐KO mice were still found to have higher glucose intolerance than WT littermates. This was not observed in mice with TERT KO in endothelial cells (EC‐TERT‐KO) or in myeloid cells (MC‐TERT‐KO) put through the same CR and HFD post‐feeding schedule. To understand the lack of metabolic rescue despite telomeres being preserved, we hypothesized that telomere‐independent effects of TERT KO in the APC lineage must be responsible. To test this, we analyzed mice with TERT KO in mature adipocytes (AD‐TERT‐KO), which do not proliferate and avoid telomere attrition. AD‐TERT‐KO mice displayed insulin resistance and reduced cold tolerance, indicating a defect in adaptive thermogenesis. Analysis of adipocytes from AD‐TERT‐KO mice indicated increased reliance on glycolysis and a decrease in oxidative metabolism due to mitochondrial dysfunction. Conversely, ectopic TERT expression in brown adipocytes induced mitochondrial oxidation and thermogenic gene expression. We conclude that metabolic dysfunction in mice with TERT KO in cells of adipose lineage is at least in part due to the loss of its non‐canonical mitochondrial function.

## 2 Materials and Methods

### 2.1 Mouse Strains

All animal studies were in compliance with the Guide and Use of Laboratory Animals and were approved by the UTHealth Animal Care and Use Committee. Mice were housed in the animal facility with a 12‐h light/dark cycle and constant temperature (22°C–24°C). The following strains characterized previously were used: *TERT* ^fl/fl^ (Liu et al. 2015), *Tie2e‐Cre* (Kano et al. 2003), LysM‐Cre (stock 004781, Jackson Laboratories), Apn‐cre (stock 010803, Jackson Laboratories), and *mT/mG* (Muzumdar et al. 2007). PCR‐based genotyping was performed as previously described (Gao et al. 2020, 2024, 2023).

### 2.2 Mouse Experiments

For TRF, 2‐month‐old mice were put into TSE PhenoMaster metabolic chambers. In the access control tab, all doors of the feeding jars were set to open at 7 pm and close at 7 am every day for 2 months so that the mice could only access the food during that time frame. The mice had free access to the water in the chambers. After TRF, the mice were transferred to regular cages and fed with HFD for 4 months. For 30% CR, 5‐week‐old (male) or 9‐month‐old (female) mice were singly housed, and 70% amount of food based on their daily food intake was fed daily for 7 weeks (male) or 1 month (female). After CR, mice were transferred to regular cages and fed with HFD until 7–8 months (male) or 14 months (female) of age. For obesity induction, mice were fed a 45 kcal% (fat) diet (Research Diets, D12451) from the cessation of TRF or CR period until terminal tissue collection. Body composition was measured by EchoMRI‐100 T (Echo Medical Systems). Glucose and insulin tolerance tests (ITT) were performed as described in our previous studies (Daquinag et al. 2021, 2022; Gao et al. 2020, 2022, 2024). Indirect calorimetry data, food intake, and locomotor activity were quantified over a 3‐day time course in Phenomaster metabolic chambers (TSE Systems). Core body temperature was determined in the rectum at 2.5 cm in depth using a MicroTherma 2K High Precision Type K Thermocouple Meter (ThermoWorks, THS‐221‐092) with RET‐3 probe (Braintree Scientific). Cold tolerance/adaptive thermogenesis was measured upon placing mice into environmental chamber IS33SD (Powers Scientific) as described. (Gao et al. 2020, 2018) Plasma lactate concentration was measured by EnzyChrom L‐Lactate Assay Kit (BioAssay Systems, cat.# ECLC‐100).

### 2.3 Tissue Analysis

SA‐βgal expression in tissue whole mounts was measured as described (Gao et al. 2020, 2018, 2024). AT formalin‐fixed paraffin‐embedded tissue sections (5 μm) were analyzed by immunofluorescence (IF) upon antigen retrieval as described (Daquinag et al. 2015, 2011; Gao et al. 2018). Upon blocking, Endomucin: R&D Systems, AF4666 (1:100), GLUT1: Mybiosourse MBS179154 (1:100), COX IV: Cell Signaling, #11967, (1:150), PDGFRβ: Abcam, ab32570 (1:100), GFP: Gene Tex, GTX26673 (1:300), and RFP: Abcam, ab34771 (1:100) primary antibodies (4°C, 12 h) were used followed by Donkey Alexa488‐conjugated (Invitrogen A11055, 1:200) or Cy3‐conjugated (Jackson ImmunoResearch 711–166‐152, 1:200) IgG. Nuclei were stained with Hoechst 33258 (Invitrogen, H3569). Fluorescence in situ hybridization (Telo‐FISH) was performed with TelC‐Cy3 as described (Gao et al. 2020, 2024). Briefly, slides treated with blocking reagent (Roche), were preheated for 5 min at 85°C followed by adding the TelC‐Cy3 probe (0.3 ng/mL) in hybridization buffer, incubation at 85°C for 10 min in a humidified chamber, and then hybridization at RT for 2 h. After 3 washes with PNA wash solution and PBS, samples counterstained with Hoechst were mounted in Fluoromount G medium. Images were acquired with a confocal Nikon AXR or Carl Zeiss upright Apotome Axio Imager Z1 microscope. Cell quantification was done using NIH ImageJ software by cell counts in 10 separate 10× fields.

### 2.4 Cell Culture and Analysis

Tissue cell suspensions were isolated as described from WAT (Gao et al. 2020, 2024) for Telo‐FISH or from ears (Gao et al. 2022) for other analyses. Minced tissue was digested in 0.5 mg/mL collagenase type I (Worthington Biochemical) and 2.5 mg/mL of dispase (Roche, 04942078001) solution under gentle agitation for 1 h at 37°C, filtered through 70 μm cell strainer (Fisherbrand, cat.# 22363548) and centrifuged at 400 *g* for 5 min to isolate the stromal/vascular pellet. Cells were plated in 8‐well chamber slides (Thermo Fisher, 154941) or 12‐well plates in DMEM/10% FBS. The Seahorse XF Cell Mito Stress Test Kit (Agilent Technologies, 103015‐100) was used to analyze mitochondrial respiration. The oxygen consumption rate (OCR) was measured upon successive treatment with 1 mM oligomycin, 1 mM FCCP, and 0.5 mM rotenone/antimycin A. TelC‐Cy3 performed as described above was done on cytospins prepare d as described (Bellows et al. 2011; Gao et al. 2024). Briefly, after 4% paraformaldehyde fixation, 5 × 10^4^ cells/200 μL were loaded into the cytology funnel (Biomedical Polymers Inc., BMP‐Cyto‐S50) with slide and spun in Cytospin 4 (Thermal Scientific) at 800 rpm for 3 min. The filter was removed from the cytology funnel/slide. The cells in a flat layer on the slides were probed with a telomere‐specific TelC‐Cy3 (PNA Bio, F1002) as described (Gao et al. 2020). MitoTracker Deep Red (Molecular Probes, M22426) was used at 0.02 mM. Immortalized brown preadipocytes (IBP) from mice were induced in a brown adipogenesis medium as described (Gao et al. 2018). Briefly, cells grown to confluence were cultured in a medium containing 50 nM insulin/0.5 mM IBMX, 1 μM dexamethasone, 1 nM 3,5,3′‐triiodothyronine (T3), and 5 μM rosiglitazone for 3 days, and 50 nM insulin with 1 nM T3 afterward. For TERT ectopic expression, mTERT‐pBabe‐puro plasmid (Addgen, cat. 36413) was transfected into Lenti‐X293T cells with packing vector in 10 cm plates using Lipofectamine 2000 (Thermo Fisher Scientific, 11668019). Virus supernatants were collected 48 h post‐transfection, filtered through a 0.45‐μm filter, and concentrated with the Lenti‐X concentrator (TakaRa Clontech, 631231). Viral infection was performed in a medium containing 8 μg/mL polybrene in 6‐well plates for 12 h. Selection with 10 μg/mL puromycin was performed for 4 days. TERT expression was confirmed by TERT mRNA expression with qPCR in six transduced clones, one of which was used.

### 2.5 DNA and RNA Analyses

DNA was extracted as described (Gao et al. 2020, 2024) by the DNeasy Blood & Tissue Kit (QIAGEN, Cat. # 69504). A quantitative real‐time PCR (qRT‐PCR) method for absolute telomere length was used as described (Gao et al. 2020; O'Callaghan and Fenech 2011). RNA was extracted using the Trizol Reagent (Life Technologies, Cat. # 15596018). Complementary DNAs were generated using a High Capacity cDNA Reverse Transcription Kit (Applied Biosystems, Cat. # 4368814). PCR reactions were performed on CFX96 Real‐Time System C1000 Touch thermal cycler (Bio‐Rad) using Q‐PCR Master Mix (Gendepot, Cat. # Q5600‐005). qPCR reactions were set at 10 μL with 5 μL of the 2XMaster Mix, 0.8 μL primers, 3 μL of cDNA (15 ng), and 1.2 μL H_2_O. All qPCRs were run in duplicates on the same thermal cycles (95°C, 5 min, 40 cycles of 15 s at 95°C, 1 min at 60°C). Expression of mouse genes was normalized to *18S RNA*. Primer sequences are provided in Table S1.

### 2.6 Statistical Analysis

Microsoft Excel and Graphpad Prism were used to graph data as mean ± SEM and to calculate *p*‐values using homoscedastic Student's *t*‐test. *p* < 0.05 was considered significant. All experiments were repeated at least twice with similar results.

## 3 Results

### 3.1 TRF‐Enabled Telomere Protection Partly Rescues APC‐TERT‐KO Metabolic Dysfunction

**FIGURE 1:**
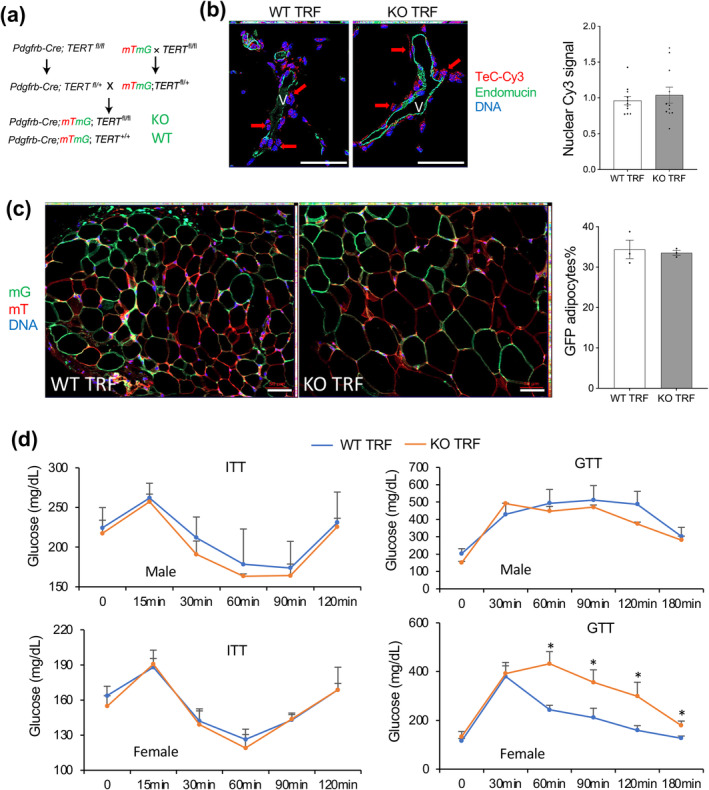
TRF effect on telomere attrition and metabolic dysfunction in APC‐TERT‐KO mice. (a) Breeding scheme to generate mice with TERT KO in APC of *Pdgfrb*+ lineage (KO), in which *Pdgfrb*+ lineage cells (fl/fl KO or +/+ WT) are labeled with the mG reporter, whereas the other cells express mT reporter. Two‐month‐old littermates were subjected to TRF for 2 months followed by 4 months of HFD feeding, after which mice were analyzed (b–d). (b) Telo‐FISH in paraffin VAT sections reveals comparable telomere length (red TelC‐Cy3 signal) in perivascular cells of WT and KO TRF males. V: Cross‐section of blood vessels revealed by endothelin IF (green). Nuclei are blue. Graph: Mean nuclear TelC‐Cy3 signal in perivascular cells. (c) GFP/RFP IF in paraffin VAT sections, identifying mG+ and mT+ cells reveals comparable frequencies of mG+ adipocytes in SAT of WT and KO TRF males. Graph: Data quantification. (d) Glucose (GTT) and insulin (ITT) tolerance test in TRF males (*n* = 6) and TRF females (*n* = 6). For all data, mean ± SEM (error bars). *p < 0.05 (two‐sided Student's *t*‐test). Scale bar: 50 μm.

First, we tested if TRF during postnatal development can help to avoid subsequent metabolic dysfunction resulting from TERT inactivation. We used a previously characterized model of TERT KO in APC of *Pdgfrb*+ lineage (APC‐TERT‐KO), in which *Pdgfrb*+ lineage cells are labeled with the mG reporter, whereas the other (TERT+ cells) are labeled with the mT reporter (Figure 1a). APC‐TERT‐KO and WT littermates were subjected to TRF starting at 2 months of age: mice had *ad libitum* (AL) access to HFD from 7 pm to 7 am and only water access for the rest of the day. During this period, total food consumption was not statistically different between AL and TRF groups (Figure S1a). However, TRF resulted in a significant delay of body mass increase at the end of the TRF phase (Figure S1b). After 2 months of TRF, all mice were placed on HFD AL for 4 months, during which the fat and lean mass of TRF mice caught‐up with that of non‐TRF controls (Figure S1b). At the age of 8 months, when APC‐TERT‐KO mice fed HFD AL from birth had been found to display comparatively higher telomere attrition and metabolic dysfunction in our previous study (Gao et al. 2020), all animals were analyzed. Analysis of inguinal SAT and gonadal VAT with the red‐fluorescent telomere probe (TelC‐Cy3) did not reveal a difference in signal intensity in the nuclei of PDGFRβ‐expressing perivascular cells of WT and APC‐TERT‐KO mice (Figure 1b). Moreover, analysis of SAT and VAT demonstrated similar frequencies of mG+ adipocytes, indicating a lack of excessive depletion of *Pdgfrb*+ lineage progenitors in APC‐TERT‐KO mice (Figure 1c). These results provide evidence that TRF slows down expedited telomere attrition caused by TERT KO and is aggravated by overnutrition and APC over‐proliferation. To determine whether this also prevents metabolic dysfunction, we subjected mice at the end of the HFD AL feeding phase to ITT and glucose tolerance test (GTT). ITT did not reveal significant differences between APC‐TERT‐KO and WT mice, indicating that insulin resistance observed for mice fed HFD AL (Gao et al. 2020) was prevented by TRF (Figure 1d). However, APC‐TERT‐KO females were found to be relatively more glucose‐intolerant than WT females (Figure 1d). Metabolic chamber analysis revealed that APC‐TERT‐KO mice had lower oxygen consumption (Figure S1b) and locomotor activity (Figure S1c). These differences in APC‐TERT‐KO mice, significant for males, were also not alleviated by TRF (Figure S1b,c). This is consistent with adipocytes in WAT of APC‐TERT‐KO mice still being hypertrophic despite TRF (Figure 1c). Combined, these data indicate that metabolic dysfunction resulting from TERT KO in APC cannot be completely prevented by telomere preservation via TRF.

### 3.2 CR‐Enabled Telomere Protection Partly Rescues APC‐TERT‐KO Metabolic Dysfunction

**FIGURE 2:**
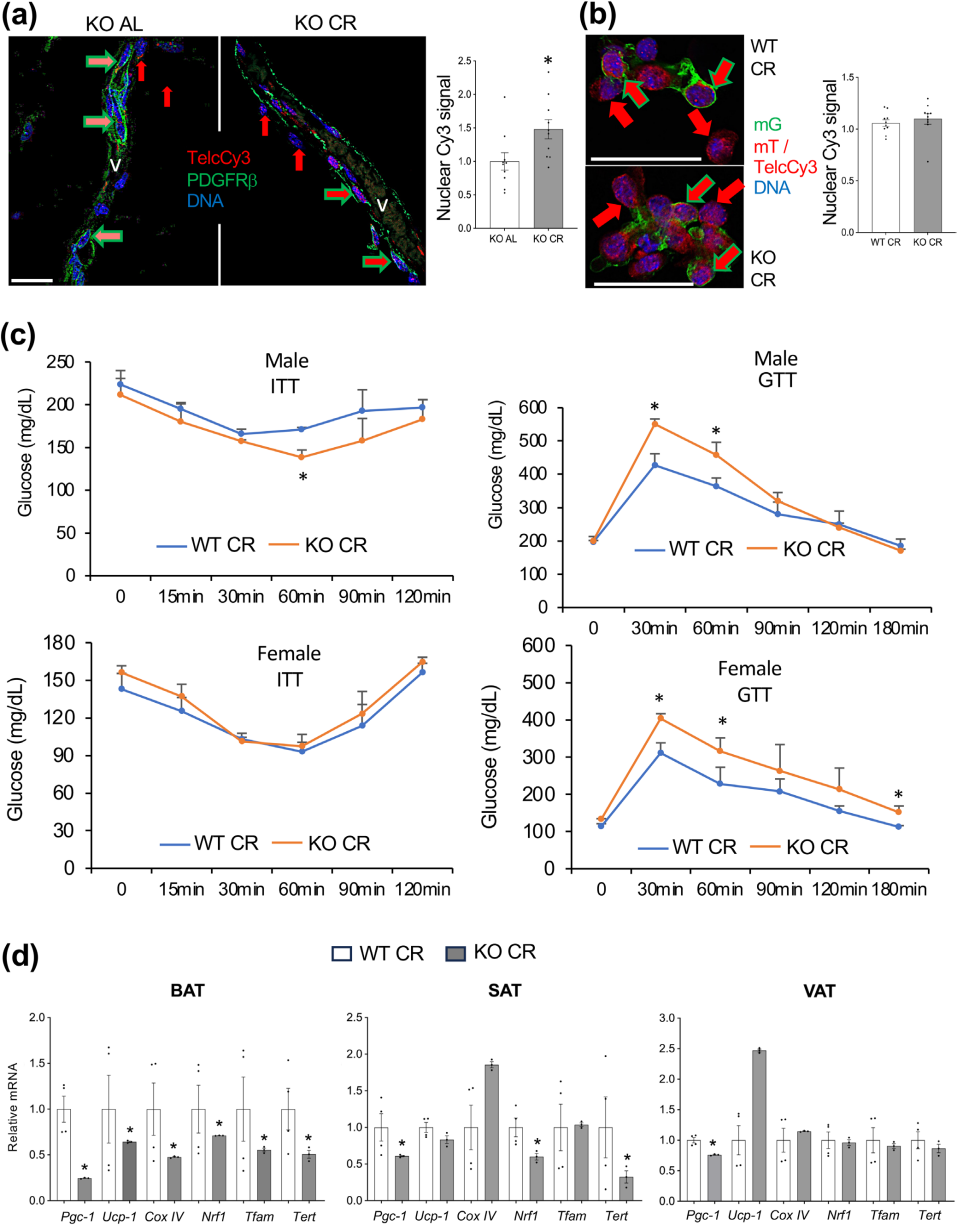
CR effect on telomere attrition and metabolic dysfunction in APC‐TERT‐KO mice. Post‐weaning, male littermates were subjected to 30% caloric restriction (CR) or were fed *ad libitum* (AL) for 2 months, followed by AL HFD feeding for all animals: (a) Telo‐FISH in paraffin VAT sections combined with PDGFRβ IF in paraffin VAT sections reveals higher telomere length (red TelC‐Cy3 signal) in perivascular PDGFRβ+ cells of KO CR males compared to KO AL males. V: Blood vessel. Nuclei are blue. Scale bar: 50 μm. Graph: Data quantifying mean nuclear TelC‐Cy3 signal in mG+ cells. (b) SAT stromal/vascular cell cytospins analyzed with TelcCy3 reveal comparable telomere lengths in mG+ cells of CR KO and CR WT males. Note that the TelC‐Cy3 signal (nuclear) in cells with cell membrane mT+ signal is comparable to that of mG+ cells. Nuclei are blue. Scale bar = 50 μm. Graph: Data quantifying mean nuclear TelC‐Cy3 signal in mG+ cells. (c) GTT and ITT in KO in males (*n* = 6) after CR at 1–2 months of age and 6 months of HFD post‐feeding (males) and in females (*n* = 6) after CR at 9–10 months of age and 3 months of HFD post‐feeding. (d) q‐RT‐PCR data (normalized to *18S* RNA) reveals lower expression of *TERT* and of mitochondrial biogenesis and metabolism genes in AT depots of CR KO vs. WT males. For all data, shown are mean ± SEM (error bars). *p < 0.05 (two‐sided Student's *t*‐test).

As a dietary approach alternative to TRF, we tested if CR during postnatal development can help to avoid metabolic dysfunction resulting from TERT inactivation. Post‐weaning, APC‐TERT‐KO and WT male littermates were subjected to 30% CR for 2 months, after which they were placed on HFD AL. At the age of 7–8 months, when APC‐TERT‐KO mice fed HFD AL from birth had been found to display comparatively higher telomere attrition and metabolic dysfunction in our previous study (Gao et al. 2020), all animals were analyzed. Analysis of inguinal SAT and gonadal VAT with Telc‐Cy3 revealed a higher signal in the nuclei of perivascular Pdgfrβ+ cells of CR KO mice compared to KO mice fed AL (Figure 2a). Analysis of stromal/vascular cell cytospins with Telc‐Cy3 confirmed that telomere lengths were comparable in WAT mT and mG cells of WT and KO CR mice (Figure 2b). These results indicate that excessive telomere erosion in APC resulting from TERT KO and over‐proliferation in AL fed‐mice (Gao et al. 2020) is suppressed by perinatal CR. Reduced oxygen consumption, observed for AL‐fed APC‐TERT‐KO mice, was not significant for CR groups but still observed (Figure S2a). CR APC‐TERT‐KO males were found to be more insulin‐sensitive (Figure 2c), which indicates that insulin resistance observed for APC‐TERT‐KO mice fed HFD AL (Gao et al. 2020) was prevented by CR. However, APC‐TERT‐KO males post‐CR and subsequent HFD feeding were found to be more glucose‐intolerant than their WT littermates (Figure 2c). In females, post‐weaning CR did improve glucose clearance, although there was no difference in the ITT profiles between the AL and CR mice (Figure S2c). However, lower glucose tolerance was still observed for APC‐TERT‐KO females subjected to CR at 9–10 months of age and HFD post‐feeding (Figure 2c). This indicated that TERT in the cells of the APC lineage has a unique function in supporting normal metabolism and that it is at least partly uncoupled from the canonical telomere‐lengthening function of TERT. To determine if this metabolic effect is unique to mice with TERT KO in *Pdgfrb*+ lineage, we used mice with TERT KO in myeloid *LysM*+ lineage (Clausen et al. 1999) or *Tie2e*+ endothelial lineage, as reported (Gao et al. 2024, 2023). Neither Tie2e‐Cre;TERT^fl/fl^ nor LysM‐Cre;TERT^fl/fl^ males displayed glucose intolerance after a CR and subsequent HFD feeding course; in fact, KO mice in both models were more insulin‐sensitive (Figure S2b). Together, these data indicate that glucose intolerance in APC‐TERT‐KO mice develops irrespective of caloric intake and, therefore, may be unrelated to telomere length.

Our studies in Tie2e‐TERT‐KO mice have established that TERT has a non‐canonical role in supporting mitochondrial biogenesis and oxidative metabolism in EC (Gao et al. 2024). To determine if this may account for the lack of phenotype rescue with TRF and CR, we measured gene expression in AT of experimental CR APC‐TERT‐KO and WT littermates at the endpoint of HFD re‐feeding. Mitochondrial biogenesis marker *Pgc1a* was significantly reduced in all AT depots of APC‐TERT‐KO mice (Figure 2d). In BAT, and to a lesser degree in SAT, this correlated with decreased expression of the downstream mitochondrial biogenesis genes *Nrf1* and *Tfam*, as well as of the oxidation gene *CoxIV* (Figure 2d). These results indicated that the mitochondrial function of TERT (Ait‐Aissa et al. 2022; Ale‐Agha et al. 2021) in metabolically active adipocytes in BAT and SAT may be critical for supporting normal oxidative metabolism.

### 3.3 Metabolic Abnormality in Mice With TERT KO in Adipocytes

**FIGURE 3:**
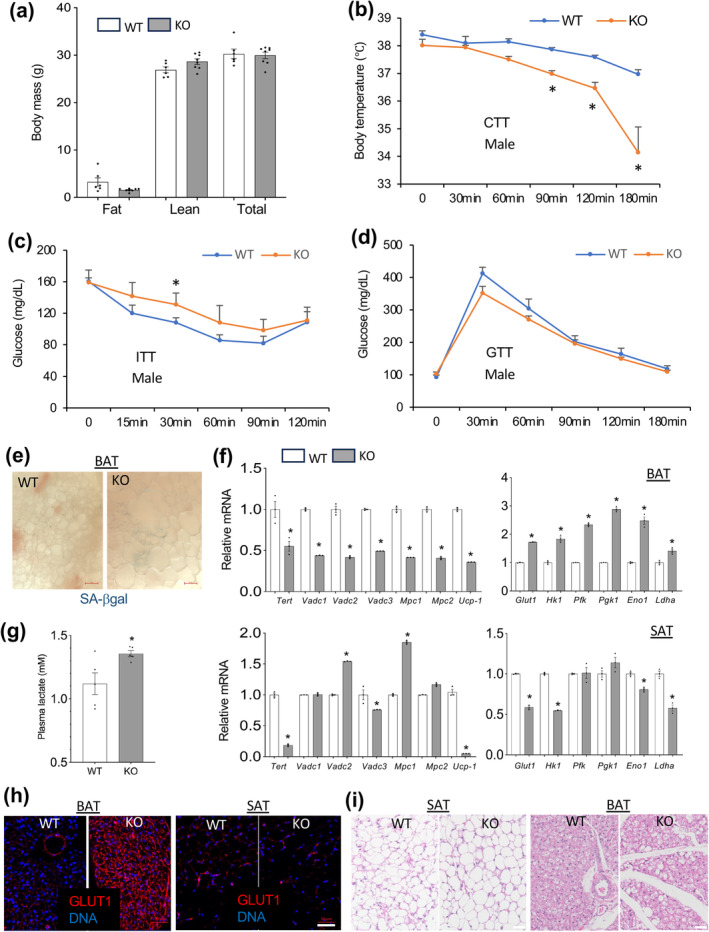
Reduced oxidative metabolism in mice with adipocyte TERT KO. Mice with TERT KO in adiponectin+ lineage cells (AD‐TERT‐KO) and WT littermates fed chow at 8–11 weeks of age were analyzed: (a) Body composition measured by EchoMRI in males. WT *n* = 7, KO *n* = 8; (b) Cold tolerance test (CTT) by body temperature measured after placement at 4°C reveals cold intolerance. WT *n* = 7, KO *n* = 8 males; (c) Intraperitoneal insulin tolerance test (ITT) in males. WT *n* = 7, KO *n* = 8; (d) Intraperitoneal glucose tolerance test (GTT) in males. WT *n* = 7, KO *n* = 8; (e) SA‐βgal expression in whole mounts of BAT of male mice: No difference between WT and KO; (f) q‐RT‐PCR data (normalized to *18S* RNA) reveals lower expression of *TERT* and of mitochondrial biogenesis and metabolism genes (left) but higher expression of glycolysis genes (right) in AT of KO males compared to WT males; (g) Increased circulating lactate levels in KO males compared to WT males; (h) IF analysis of GLUT1 expression in paraffin sections showing its induction in BAT of KO males compared to WT males; (i) H&E histology of paraffin sections revealing larger lipid droplets in BAT of KO males compared to WT males. Scale bar: 50 μm. For all data, shown are mean ± SEM (error bars). *p < 0.05 (two‐sided Student's *t*‐test).

TERT KO in Pdgfrb+ lineage cells results in adipocyte derivatives of these cells also being TERT‐deficient. Thus, we considered the possibility that the incomplete phenotype rescue by CR is due to the function of TERT in adipocytes. *Ucp1* expression was also lower in BAT of APC‐TERT‐KO mice, suggesting that uncoupled thermogenesis is undermined (Figure 2d). *Ucp1* is expressed in mature brown adipocytes (Cannon and Nedergaard 2004; Kajimura, Spiegelman, and Seale 2015), consistent with the possibility of altered cell function post‐differentiation. We therefore created mice with TERT KO specifically in mature adipocytes (AD‐TERT‐KO) based on Cre expression in cells expressing Adiponectin (*Apn*) (Shook et al. 2014). Analysis of AD‐TERT‐KO and WT littermates did not reveal a significant difference in fat or lean body mass (Figure 3a) or energy expenditure (Figure S3a). While AD‐TERT‐KO males had lower locomotor activity than WT littermates, the opposite trend was observed for AD‐TERT‐KO females (Figure S3b). Consistent with *Ucp1* expression reduction in adipocytes, AD‐TERT‐KO males displayed a significant cold intolerance (Figure 3b), whereas no significant differences were seen in KO and WT females (Figure S3c). AD‐TERT‐KO mice were found to be more insulin‐resistant than WT mice, which was the case for males (Figure 3c) and females (Figure S3d). However, glucose tolerance was normal in both males (Figure 3d) and females (Figure S3e).

To determine if diet can influence the metabolism of AD‐TERT‐KO mice, we fed separate cohorts HFD for 6 months. As for chow, body weights were comparable for AD‐TERT‐KO and WT littermates on HFD (Figure S4a). There was only a trend for lower cold tolerance in AD‐TERT‐KO males (Figure S4b). Like chow‐fed mice, AD‐TERT‐KO males were glucose‐intolerant (Figure S4c) and slightly more insulin‐resistant (Figure S4d) than WT on HFD. Metabolic chamber analysis did not reveal significant differences in energy expenditure (Figure S4e), substrate utilization (Figure S4f), or food intake (Figure S4g) between AD‐TERT‐KO and WT males fed HFD. AD‐TERT‐KO females on HFD, like males, did not display a difference in body composition (Figure S4i) but were more insulin‐resistant than WT (Figure S4j). Decreased oxidative metabolism (Figure S4k) or locomotor activity (Figure S4l) was not observed in AD‐TERT‐KO females fed HFD. The less‐pronounced thermogenesis phenotype observed for males on HFD suggests that a higher carbohydrate/lipid ratio in the diet predetermines metabolic changes resulting from adipocyte TERT‐KO.

The cold intolerance in AD‐TERT‐KO males, coupled with metabolic dysfunction, indicated that TERT plays a role in BAT thermogenesis. To establish the mechanism of thermogenic defect, we analyzed mouse tissues. Higher levels of cell senescence, previously reported for APC‐TERT‐KO (Gao et al. 2020), were not detected in BAT of AD‐TERT‐KO mice according to SA‐βgal staining (Figure 3e). However, total BAT gene expression analysis revealed that, concordantly with *TERT* and *Ucp1*, mitochondrial oxidation markers *Vdac1*, *Vdac2*, *Vdac3*, *Mcp1*, and *Mcp2* were significantly decreased in AD‐TERT‐KO mice (Figure 3f). In contrast, glycolysis marker genes *Glut1*, *Hk1*, *Pfkl*, *Pgk1*, *Eno1*, and *Ldha* were significantly increased in AD‐TERT‐KO BAT (Figure 3f). This pattern was also observed for SAT of AD‐TERT‐KO mice, although it was less pronounced (Figure 3f). Consistent with glycolysis marker upregulation, analysis of mouse plasma demonstrated higher lactate concentration in AD‐TERT‐KO mice fed chow (Figure 3g). Interestingly, on HFD circulating lactate was not higher in AD‐TERT‐KO mice, indicating that preferential lipid utilization overrides glycolysis (Figure S4h). Induction of GLUT1 expression in AD‐TERT‐KO at the protein level was demonstrated by IF in BAT (Figure 3h). BAT of AD‐TERT‐KO animals also displayed a trend for the accumulation of larger lipid droplets (Figure 3i). These results suggested that TERT supports normal oxidative metabolism in brown adipocytes.

### 3.4 TERT Is Required for Mitochondrial Biogenesis and Respiration in Brown Adipocytes

**FIGURE 4:**
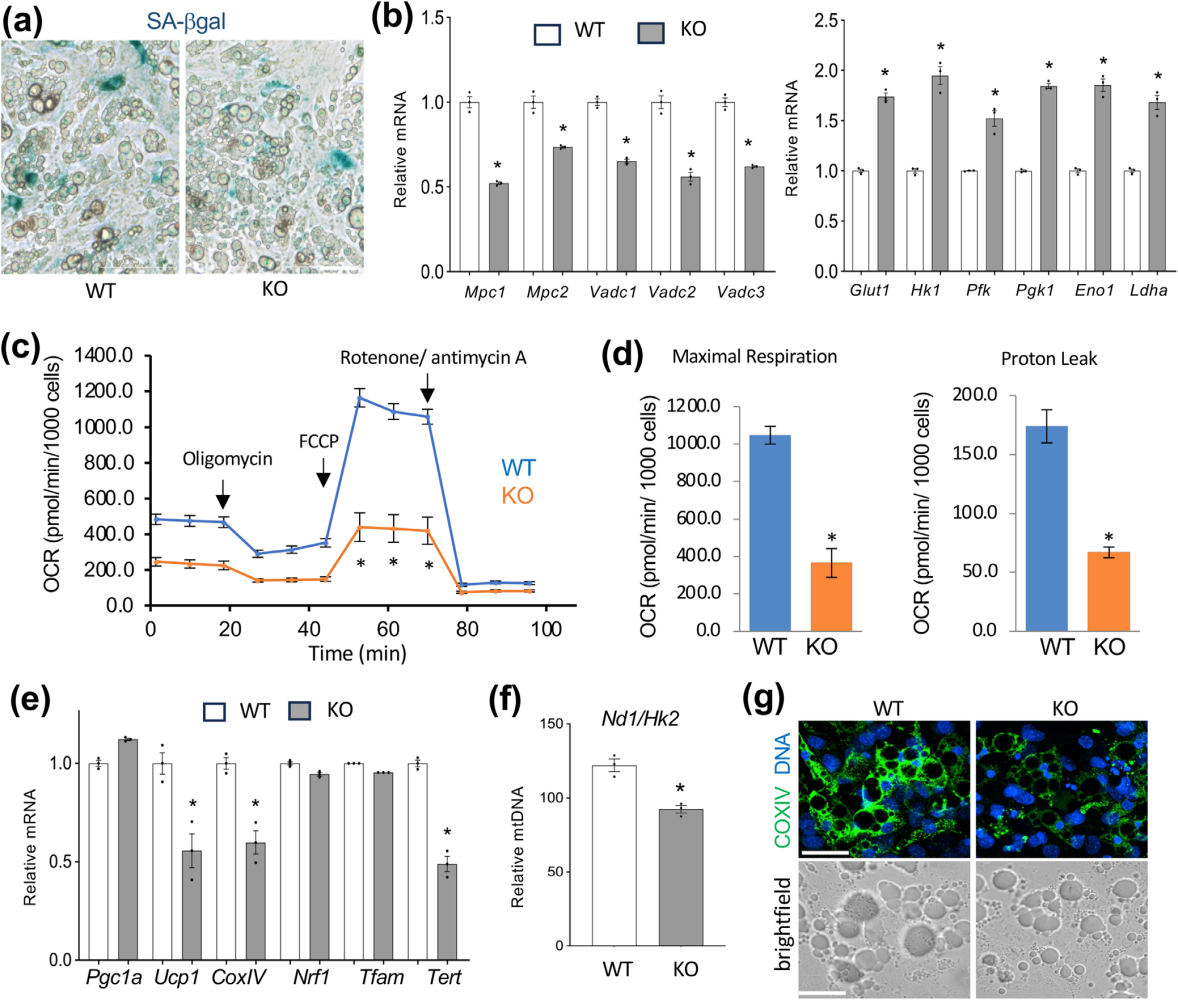
TERT is required for mitochondrial function in brown adipocytes: (a) Ear fibroblasts from 2‐month‐old WT and AD‐TERT‐KO littermates induced for brown adipogenesis display comparable lipid droplet accumulation and background SA‐βgal expression; (b) q‐RT‐PCR data (normalized to *18S* RNA) reveals lower expression of mitochondrial metabolism genes (left) but higher expression of glycolysis genes (right) in AD‐TERT‐KO brown adipocytes; (c) XF Cell Mito Stress assay used to analyze mitochondrial respiration. Oxygen consumption rate (OCR), measured upon successive treatment with oligomycin, FCCP (carbonyl cyanide‐*p*‐trifluoromethoxyphenylhydrazone), and rotenone/antimycin A, demonstrates lower basal and induced mitochondrial function in TERT KO adipocytes. (d) Maxima OCR and oligomycin‐resistant OCR, reflecting ATP‐uncoupled respiration. (e) q‐RT‐PCR data (normalized to *18S* RNA) reveals lower expression of *TERT* and of mitochondrial biogenesis and thermogenesis genes in AD‐TERT‐KO brown adipocytes. (f) Relative mitochondrial DNA, based on the ratio of *ND1* to *HK2* amplification by qPCR, reveals lower mitochondrial numbers in AD‐TERT‐KO brown adipocytes. (g) Cox IV immunofluorescence showing reduced mitochondrial content in KO adipocytes. Brightfield images revealing adipocyte lipid droplets are shown below. Scale bar: 50 μm. For all data, shown are mean ± SEM (error bars). *p < 0.05 (two‐sided Student's *t*‐test).

Progenitor cells from AD‐TERT‐KO and WT littermates differentiated into adipocytes equally well upon induction of brown adipogenesis and SA‐βgal staining did not reveal senescence induction above the level seen for WT cells (Figure 4a). Expression of senescence markers *p16*, *p21*, and *p53* was also not induced (Figure S5a). However, AD‐TERT‐KO adipocytes had higher expression of genes coding for cytokines TNFα, IL‐1, and IL‐6 (Figure S5a). As in BAT, the expression of mitochondrial oxidation markers *Vdac1*, *Vdac2*, *Vdac3*, *Mcp1*, and *Mcp2* was significantly decreased in TERT‐KO adipocytes (Figure 4b). Conversely, a compensatory induction of glycolysis markers *Glut1*, *Hk1*, *Pfk1*, *Pgk1*, *Eno1*, and *Ldha* was observed in TERT‐KO adipocytes (Figure 4b). In accordance with these data, Seahorse analysis demonstrated that TERT‐KO adipocytes had reduced mitochondrial respiration (Figure 4c) and proton leak (Figure 4d). Consistent with these observations, gene expression analysis demonstrated that TERT KO adipocytes had a significantly lower expression of *Ucp1*, mitochondrial biogenesis makers *Nrf1* and *Tfam*, as well as respiratory chain factor *CoxIV*, compared to WT adipocytes (Figure 4e). To establish the mechanism responsible for this, we quantified mitochondrial DNA by qPCR assay measuring the ratio of mitochondrial DNA amplification relative to autosomal DNA (*mt‐Nd1/Hk2*). This revealed that TERT KO adipocytes have significantly fewer mitochondria (Figure 4f). Reduced mitochondrial activity was further confirmed by CoxIV IF (Figure 4g) and staining adipocytes with Mitotracker Red (Figure S5b). In brown adipocytes differentiated in culture, TERT KO did not have an effect on mitochondrial DNA integrity (Figure S5c). However, in mice fed HFD for 8 months, there was a trend for higher mitochondrial DNA damage (Figure S5d).

### 3.5 Ectopic TERT Induces Mitochondrial Biogenesis and Respiration in Brown Adipocytes

**FIGURE 5:**
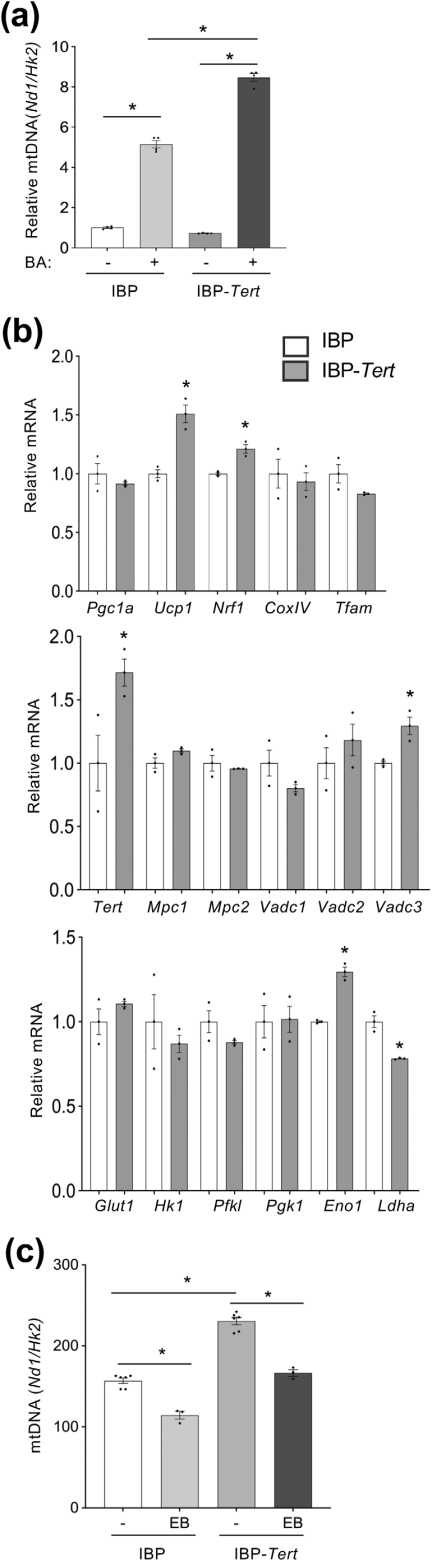
TERT is a driver of mitochondrial function in brown adipocytes. (a) Relative mitochondrial DNA, based on the ratio of *Nd1* to *Hk2* amplification by qPCR, reveals higher mitochondrial numbers in immortalized brown preadipocytes (IBP) induced for brown adipogenesis. IBP‐*Tert*: IBP transduced with mouse *Tert*. Note that IBP‐*Tert* adipocytes have higher mitochondrial content than IBP adipocytes. (b) q‐RT‐PCR data (normalized to *18S* RNA) reveals higher expression of Tert and of mitochondrial biogenesis, and metabolism, concomitant with reduced *Ldha* expression in AD‐TERT‐KO brown adipocytes. (c) IBP brown adipocytes were treated with 25 mM ethidium bromide (EB) for 1 h in 10% FBS DMEM. Mitochondrial DNA was analyzed based on *Nd1/Hk2* amplification by qPCR. Control *n* = 2 with triplicates, EB *n* = 1 with triplicates. For all data, shown are mean ± SEM (error bars). *p < 0.05 (two‐sided Student's *t*‐test).

Finally, we used mouse IBP transduced with a mouse *Tert* expression vector. Upon brown adipogenesis induction, IBP‐*Tert* adipocytes were found to have higher mitochondrial content than the parental IBP adipocytes (Figure 5a). Ectopic *Tert* expression resulted in a higher expression of *Ucp1* (Figure 5b). Some of the mitochondrial oxidation markers, *Vdac2, Vdac3*, and *Mcp1*, were concordantly elevated (Figure 5b). Conversely, there was a reduction of glycolysis markers *Hk1, Pfk1*, and *Ldha* (Figure 5b). Combined these results indicate that TERT is not only necessary but is also sufficient to stimulate mitochondrial biogenesis, oxidative metabolism, and thermogenesis in brown adipocytes. However, ectopic *Tert* expression did not protect mitochondria from ethidium bromide damage, as assessed by mitochondrial DNA reduction measured by qPCR (Figure 5c).

## 4 Discussion

TERT is a pivotal gero‐protective protein that enables telomere maintenance, mitigates genotoxic stress, and supports mitochondrial function (Romaniuk et al. 2019; Sahin and Depinho 2010). In humans, *TERT* is expressed in stem cells but turned off in somatic cells, which permits telomere erosion and cell aging. Cell senescence, an underpinning of aging, is a state of cell‐cycle arrest and pro‐inflammatory cytokine upregulation (Fossel et al. 2022; Yousefzadeh et al. 2021), is partly responsible for metabolic changes leading to age‐related diseases (Fossel et al. 2022). Importantly, TERT not only lengthens telomeres to prevent their attrition (Blackburn, Greider, and Szostak 2006; Blasco 2007) but is also implicated in protection from genotoxic stress (Sahin and Depinho 2010). TERT also has genome‐wide telomere‐independent effects on gene expression and cell metabolism (Mojiri et al. 2021). Resistance of cells to senescence relies on mitochondrial function (Wiley et al. 2016). There is a growing body of evidence that the mitochondrial function of TERT is important in this process (Ait‐Aissa et al. 2022; Ale‐Agha et al. 2021; Beyer and Norwood Toro 2018; Sahin et al. 2011). In our recent study, TERT KO in EC was found to trigger telomere‐independent mitochondrial dysfunction, resulting in cell senescence and aging‐associate phenotypes: tissue hypoxia and hypotrophy, cognitive impairment, and muscle endurance loss (Gao et al. 2024). We have found that the extranuclear function of TERT, supporting mitochondrial biogenesis, and oxidative metabolism was responsible for these phenotypes (Gao et al. 2024). We have also reported that inactivation of TERT in APC expedites their telomere attrition, the onset of replicative senescence, and metabolic disease in mice (Gao et al. 2020). However, whether the loss of canonical or telomere‐independent function of TERT was responsible for the premature onset of AT dysfunction has remained unclear.

Our previous studies indicated that replicative senescence of APC is promoted by their overnutrition‐induced over‐proliferation (Gao et al. 2020; Lin et al. 2024). Here, we tested if caloric intake can be used to control APC exhaustion and metabolic decline. We demonstrate that dietary interventions do not completely rescue the metabolic defect in mice with APC TERT KO despite the suppression of telomere attrition (Figure S6). Our results indicate that metabolic abnormalities in APC‐TERT‐KO models result to an extent from the dysfunction of brown adipocytes, derived from APC, which lack the mitochondrial TERT function supporting respiration and thermogenesis in these KO mice. Previously reported genomic analysis of APC from APC‐TERT‐KO mice (Gao et al. 2020) revealed decreased expression of VDAC and MPC pyruvate transporters. With reduced mitochondrial function, the citrate cleavage pathway, supplying cytosolic acetyl‐CoA and NADPH for fatty acid synthesis, is limited, making lipogenesis inefficient. This likely accounts for the compensatory induction of lipogenesis genes observed for APC‐TERT‐KO adipocytes (data not shown). The gene expression changes indicating a switch to glycolysis, pronounced in BAT were milder in SAT and negligible in VAT. This is consistent with TERT importance being high in brown and beige adipocytes, rich in mitochondria, but not in white adipocytes, which have lower oxidative activity. Glycolytic brown adipocytes have been previously reported to arise in BAT and beige AT (Chen et al. 2019; Jun et al. 2020). One of the markers reported for glycolytic AT in these studies, GABPa, was indeed increased upon TERT KO (data not shown). Consistent with our results from mice with TERT KO in EC (Gao et al. 2024), TERT KO brown adipocytes were found to have a decrease in the expression of mitochondrial biogenesis genes and in mitochondrial content. Therefore, it is likely that TERT supports oxidative metabolism mainly through mitochondrial biogenesis regulated at the transcription level in the nucleus. Indeed, consistent with previously reported epigenetic effect of TERT (Mojiri et al. 2021), the genes we found to be modulated by TERT levels are chromosomal. However, in human cell models, overexpressed TERT has also been shown to localize to mitochondria, bind to mitochondrial DNA, and protect it from damage (Haendeler et al. 2009). While our data indicate that mouse TERT has a similar function in protecting mitochondrial DNA in BAT from overnutrition‐induced damage, ectopic *Tert* expression did not protect mitochondrial DNA from damage in cultured cells. This suggests possible species‐specific differences in TERT function.

We used two different approaches to dietary intervention: 2 months of TRF starting at 2 months of age versus 2 months of continuous 30% CR starting at 4 weeks of age in APC‐TERT‐KO mice. Whereas overall food consumption was reduced only for CR, both regimens were found to result in reduced telomere attrition compared to AL‐fed mice. This suggests that restoration of diurnal cycling of APC proliferation, abrogated by HFD feeding (Ribas‐Latre et al. 2021), is sufficient to prevent the APC pool exhaustion caused by continuous excessive replication. The overall metabolic phenotypes still observed in APC‐TERT‐KO mice subjected to TRF and CR were similar. The exception is that glucose intolerance, still observed for APC‐TERT‐KO males after CR, was not observed after TRF. This could potentially be explained by the slightly different timing of the two dietary interventions. The differences in telomere‐independent phenotypes in APC‐TERT‐KO and AD‐TERT‐KO mice are particularly interesting. We did not observe significant cold intolerance in APC‐TERT‐KO mice even fed AL (Gao et al. 2020). Also, while APC‐KO mice post‐CR become insulin‐sensitive but remain glucose‐intolerant, AD‐KO mice are glucose‐tolerant but insulin‐resistant. These distinctions are likely due to TERT loss in all *Pdgfrb*+ lineage cells not only in AT but also in other organs of APC‐TERT‐KO mice. The resulting perivascular cell dysfunction in the liver, pancreas, skeletal muscle, and brain collectively accounts for their phenotype. Moreover, we have reported that TERT KO in EC leads to systemic cell senescence induction spreading beyond EC to other cell types (Gao et al. 2024). Such juxtracrine senescence is also likely to take place in tissues of APC‐TERT‐KO mice. Interestingly, we did not observe the induction of senescence markers in AT of AD‐TERT‐KO mice, although the expression of inflammatory cytokines was increased. The extent of senescence induction due to TERT KO in different cell types likely also contributes to the phenotype nuances in the different models. There are known differences in AT physiology between men and women, which remain incompletely understood (Karastergiou and Fried 2017; Rosen and Spiegelman 2014). Consistent with that notion, there were sex‐specific differences observed in the TERT KO models previously (Gao et al. 2020, 2024) and also in this study. While they are likely to be due to the effect of female steroid hormones, the lack of dependency of thermogenic adipocytes on TERT function in females remains to be further investigated.

## 5 Conclusions

Our study provides evidence that *Tert* in adipocytes plays an important role in maintaining their mitochondrial oxidation, supporting healthy metabolism. This study, based on two novel mouse models, has some limitations. First, TRF was performed early, during postnatal development. Because growth of all tissues is ongoing at that stage, there was an effect on lean body mass. As a result, the subsequent HFD challenge potentially made these mice, with less skeletal muscle tissue than AL‐fed controls, more metabolically challenged. Also, the dietary interventions were performed for only 2 months, which was a time frame expected to be sufficient to affect the APC pool exhaustion but not long enough to further affect normal physiology. It remains to be determined what effects on telomeres' different approaches to CR performed later in life may have. Finally, the KOs were based on constitutive Cre lines, which are not suitable for studying the short‐term effects of Cre inactivation. With the repercussions of even constitutive KOs being mild here and in the previous study (Gao et al. 2020), it is unlikely that we would have detected a phenotype upon inducible TERT inactivation. Nevertheless, the results from the expression of ectopic *Tert* during brown adipocyte differentiation support our conclusions about the TERT's role in mitochondrial biogenesis and function. Our results showing that ectopic TERT expression in adipocytes induces mitochondrial oxidation and thermogenic gene expression suggest adipocyte TERT gene therapy as an approach to mitigate aging‐associated metabolic dysfunction.

## Author Contributions

M.G.K. and K.E.‐M. conceived and directed the project. Z.G., and Y.Y. performed the experiments and statistical analysis. M.G.K. wrote the manuscript, K.E.‐M. and Z.G. reviewed and revised the manuscript. All authors discussed and approved the final manuscript.

## Conflicts of Interest

The authors declare no conflicts of interest.

## Supporting information


Data S1.

## Data Availability

The data that support the findings of this study are available from the corresponding author upon request.
