# Supplementary material for: Caloric Restriction and Telomere Preservation in TERT Knockout Adipocyte Progenitors Does Not Rescue Mice From Metabolic Dysfunction due to a TERT Function in Adipocyte Mitochondria
Source: Aging Cell. 2025 Feb 11;24(3):e14499. doi: 10.1111/acel.14499 (PMC11896407; doi:10.1111/acel.14499)
Supplement: Supplementary file 1 — Data S1. [file ACEL-24-e14499-s001.pdf]

Tert forward Integrated DNA Technologies 5′- GGATTGCCACTGGCTCCG -3′
Tert reverse Integrated DNA Technologies 5′-TGCCTGACCTCCTCT TGTGAC-3′;
Hk1 forward Integrated DNA Technologies 5’-CATTGTCTCCTGCATCTCCGA-3’
Hk1 reverse Integrated DNA Technologies 5’-ATTCCGCAATCTAGGCTCGTC-3’
Pfkl forward Integrated DNA Technologies 5’-AACGCTGCAATGGAGAGTTGT-3’
Pfkl reverse Integrated DNA Technologies 5’-TCAAAGACGTAGGCAGCATCG-3’
18S RNA forward Integrated DNA Technologies 5′-AAGTCCCTGCCCTTTGTACACA-3′
18S RNA reverse Integrated DNA Technologies 5′-GATCCGAGGGCCTCACTAAAC-3′
Eno1 forward Integrated DNA Technologies 5’-AGATCCCTTTGACCAGGACGA-3’
Eno1 reverse Integrated DNA Technologies 5’- AGCCTTGG CAATCCGCTTA-3’
Pgk1 forward Integrated DNA Technologies 5’-TTTGGACAAGCTGGACGTGAA-3’
Pgk1 reverse Integrated DNA Technologies 5’-GCTTGGAACAGCAGCCTTGAT-3’
Ldha forward Integrated DNA Technologies 5’-ACGCAGACAAGGAGCAGTGGAA -3’
Ldha reverse Integrated DNA Technologies 5’- ATGCTCTCAGCCAAGTCTGCCA-3’
Glut1 forward Integrated DNA Technologies 5’- TCTTCGAGAAGGCAGGTGT -3’
Glut1 reverse Integrated DNA Technologies 5’- TCCAGCTCGCTCTACAACAA -3’
Pgc1 forward Integrated DNA Technologies 5’- GCACCAGAAAACAGCTCCAAG-3’
Pgc1 reverse Integrated DNA Technologies 5’- CGTCAAACACAGCTTGACAGC-3’
Ucp1 forward Integrated DNA Technologies 5′-TCTCAGCCGGCTTAATGACTG-3′
Ucp1 reverse Integrated DNA Technologies 5′-GGCTTGCATTCTGACCTTCAC-3′
Cox IV forward Integrated DNA Technologies 5'-CTGCCCGGAGTCTGGTAATG-3'
Cox IV reverse Integrated DNA Technologies 5'-CAGTCAACGTAGGGGGTCATC-3'
Nrf1 forward Integrated DNA Technologies 5’-GATGCTTCAGAACTGCCAACCA-3’
Nrf1 reverse Integrated DNA Technologies 5’-GGTCATTTCACCGCCCTGTAAC-3’
Tfam forward Integrated DNA Technologies 5’-TGAAGCTTGTAAATGAGGCTTGGA-3’
Tfam reverse Integrated DNA Technologies 5’- CGGATCGTTTCACACTTCGAC-3’
Nd1 forward Integrated DNA Technologies 5’- CTAGCAGAAACAAACCGGGC-3’
Nd1 reverse Integrated DNA Technologies 5’- CCGGCTGCGTATTCTACGTT-3’
Hk2 forward Integrated DNA Technologies 5’-GCCAGCCTCTCCTGATTTTAGTGT-3’
Hk2 reverse Integrated DNA Technologies 5’-GGGAACACAAAAGACCTCTTCTGG-3’
Mpc1 forward Integrated DNA Technologies 5’- CAAGGACTTCCGGGACTATC-3’
Mpc1 reverse Integrated DNA Technologies 5’- CATCCGCCCACTGATAATCTC-3’
Mpc2 forward Integrated DNA Technologies 5’- CCGACTCATGGATAAAGTGGAG-3’
Mpc2 reverse Integrated DNA Technologies 5’- CTAGTCCAGCACACACCAAT-3’
Vdac1 forward Integrated DNA Technologies 5’- AGTGACCCAGAGCAACTTCGCA-3’
Vdac1 reverse Integrated DNA Technologies 5’- CAGGCGAGATTGACAGCAGTCT-3’
Vdac2 forward Integrated DNA Technologies 5’- TCGGCAAAGCTGCCAGAGACAT-3’
Vdac2 reverse Integrated DNA Technologies 5’- GTCTCCAAGGTCCCGCTAACTT-3’
Vdac3 forward Integrated DNA Technologies 5’- GCCTTTGAAGGTTGGCTTGCTG-3’
Vdac3 reverse Integrated DNA Technologies 5’- GAGCCTCCAAACTCAGTGCCAT-3’

Table S1. Sequences of primers used for PCR.

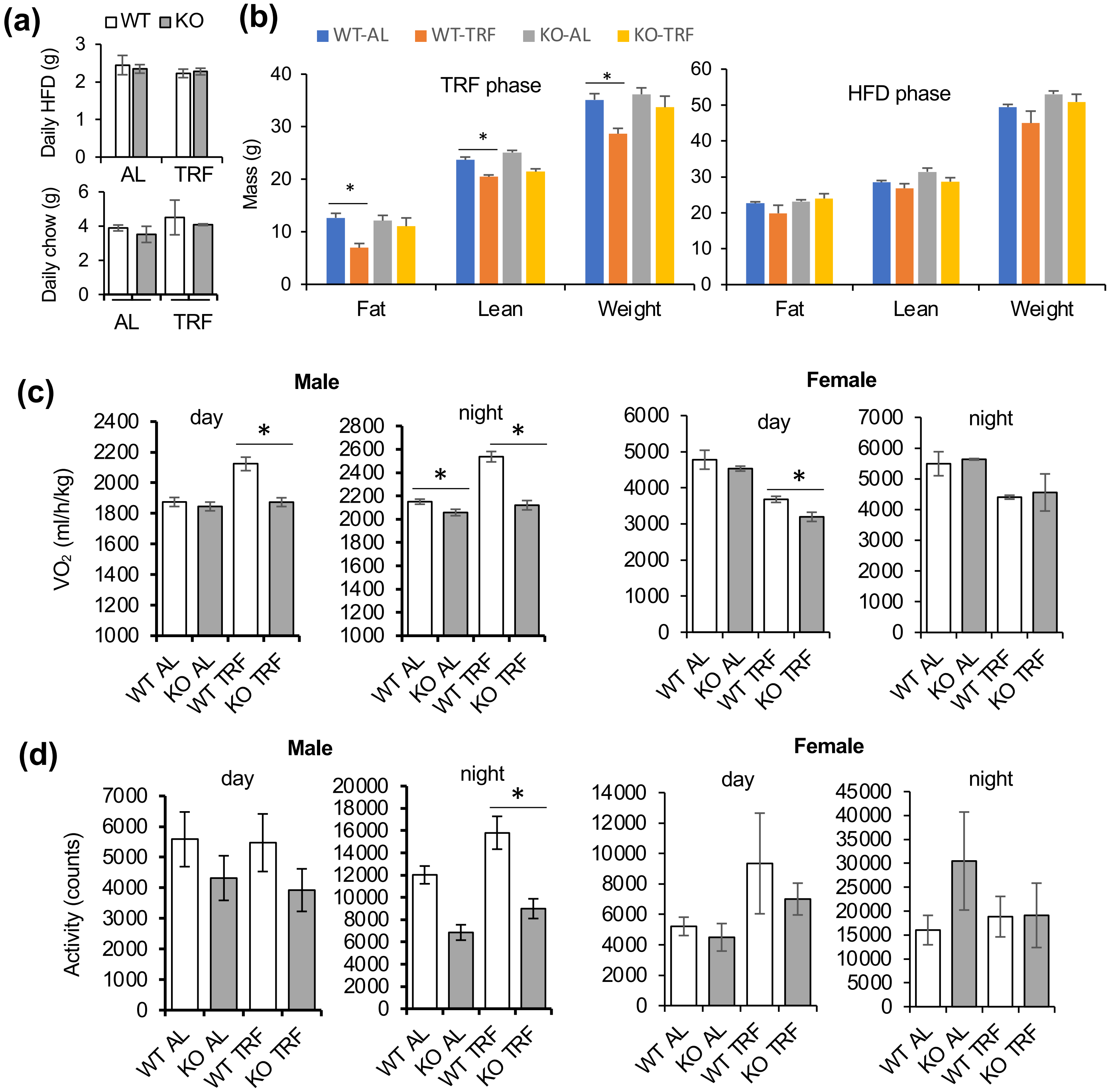

Figure S1. Time-restrictive feeding prevents telomere attrition in APC-TERT-KO mice and partly rescues metabolic dysfunction

(a) Daily food consumption by mice during the TRF period (mean over a 3-day period).

(b) Body composition measured by EchoMRI demonstrates that body mass gain is delayed in both WT and APC-TERT-KO males at the end of the TRF phase and catches up at the end of the HFD feeding phase.

(c) Calorimetric measurement of VO_2 in WT and APC-TERT-KO males at the end of the HFD feeding phase during day and night cycles (n = 3). and females

(d) Measurement of locomotor activity in WT and APC-TERT-KO males and females at the end of the HFD feeding phase during day and night cycles (n = 3).

For all data, shown are mean+/- SEM (error bars). *p < 0.05 (two-sided Student's t-test).

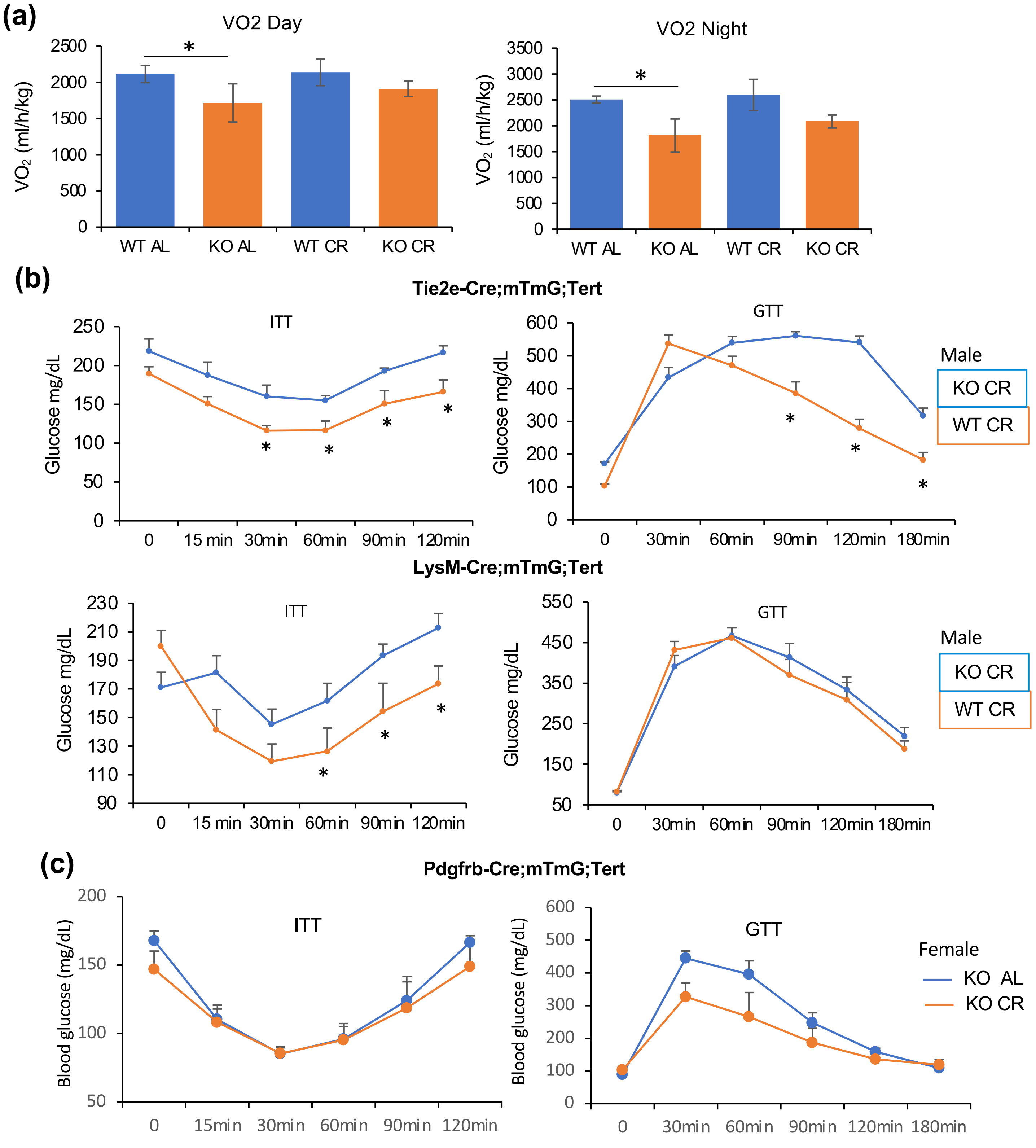

Figure S2. CR effect on mice with TERT-KO in APC, endothelial and myeloid cells

(a) Calorimetric measurement of VO_2 in WT and APC-TERT-KO males post-CR/AL feeding at the end of the HFD feeding phase during day and night cycles.

For all data, shown are mean+/- SEM (error bars) for n = 6.

(b) EC-TERT-KO and WT males, created by crossing Tie2e-Cre and $TERT^{fl/fl}$ mice (top) and Monocyte-TERT-KO and WT males, created by crossing LysM-Cre and $TERT^{fl/fl}$ mice (bottom) were subjected to 30% CR at 2 months of age, followed by HFD feeding. IP ITT and GTT were performed at the end of the HFD phase.

(c) APC-TERT-KO females AL-fed or subjected to 30% CR at 2 months of age, followed by HFD feeding. IP ITT and GTT were performed at the end of the HFD phase.

*$p < 0.05$ (two-sided Student's t-test).

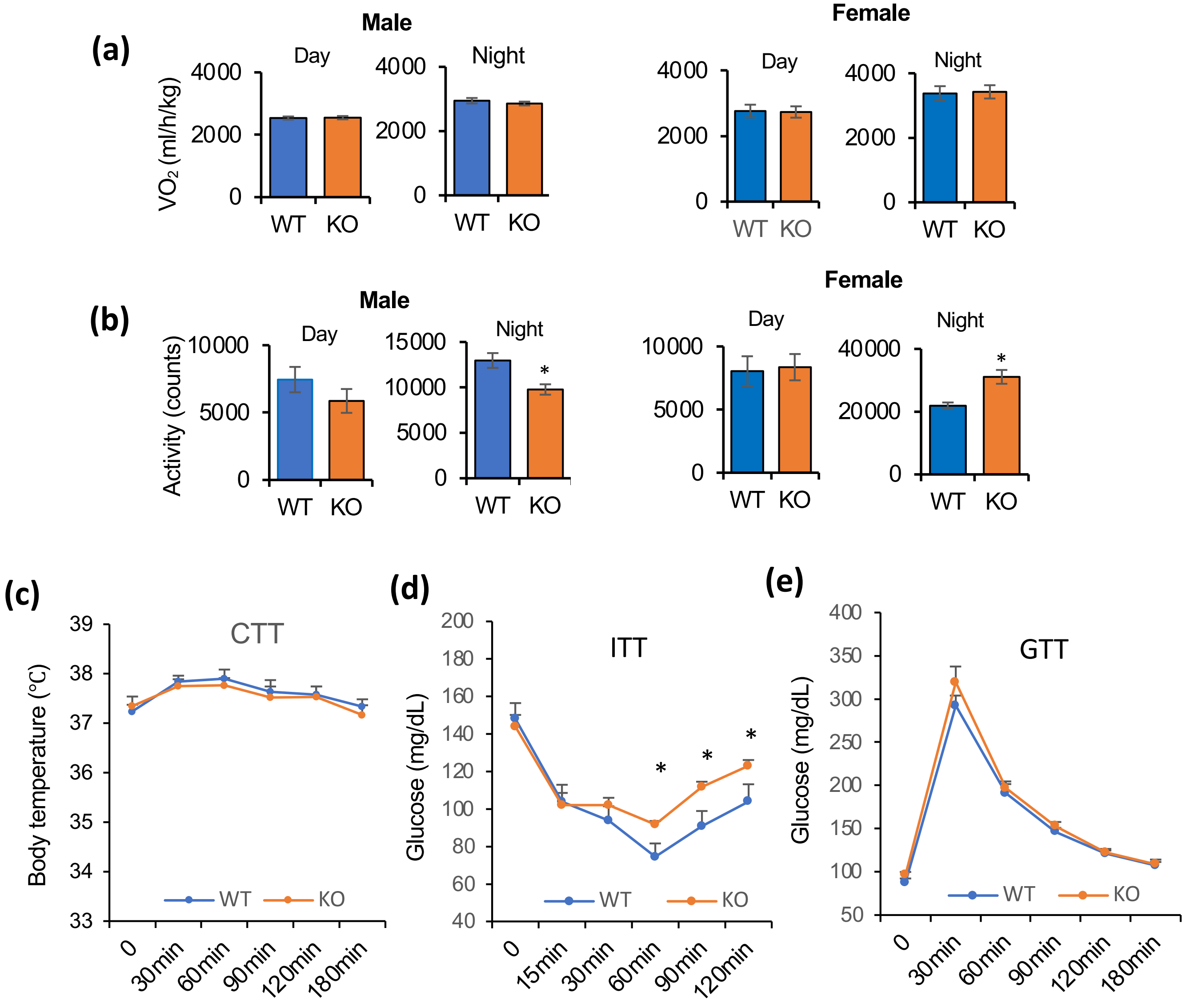

Figure S3. Metabolism in mice with adipocyte TERT KO fed chow

Mice with TERT KO in Adiponectin+ lineage cells (AD-TERT-KO) and WT littermates fed chow at 8-11 weeks of age were analyzed.

(a) Calorimetric measurement of VO_2 in WT and AD-TERT-KO males and females during day and night cycles (n = 6).

(b) Measurement of locomotor activity in WT and AD-TERT-KO males and females during day and night cycles (n = 6).

(c) Cold tolerance test (CTT) in females (n = 6).

(d) Intraperitoneal insulin tolerance test (ITT) in females (n = 6).

(e) Intraperitoneal glucose tolerance test (GTT) in females (n = 6).

For all data, shown are mean+/- SEM (error bars). *$p < 0.05$ (two-sided Student's t-test).

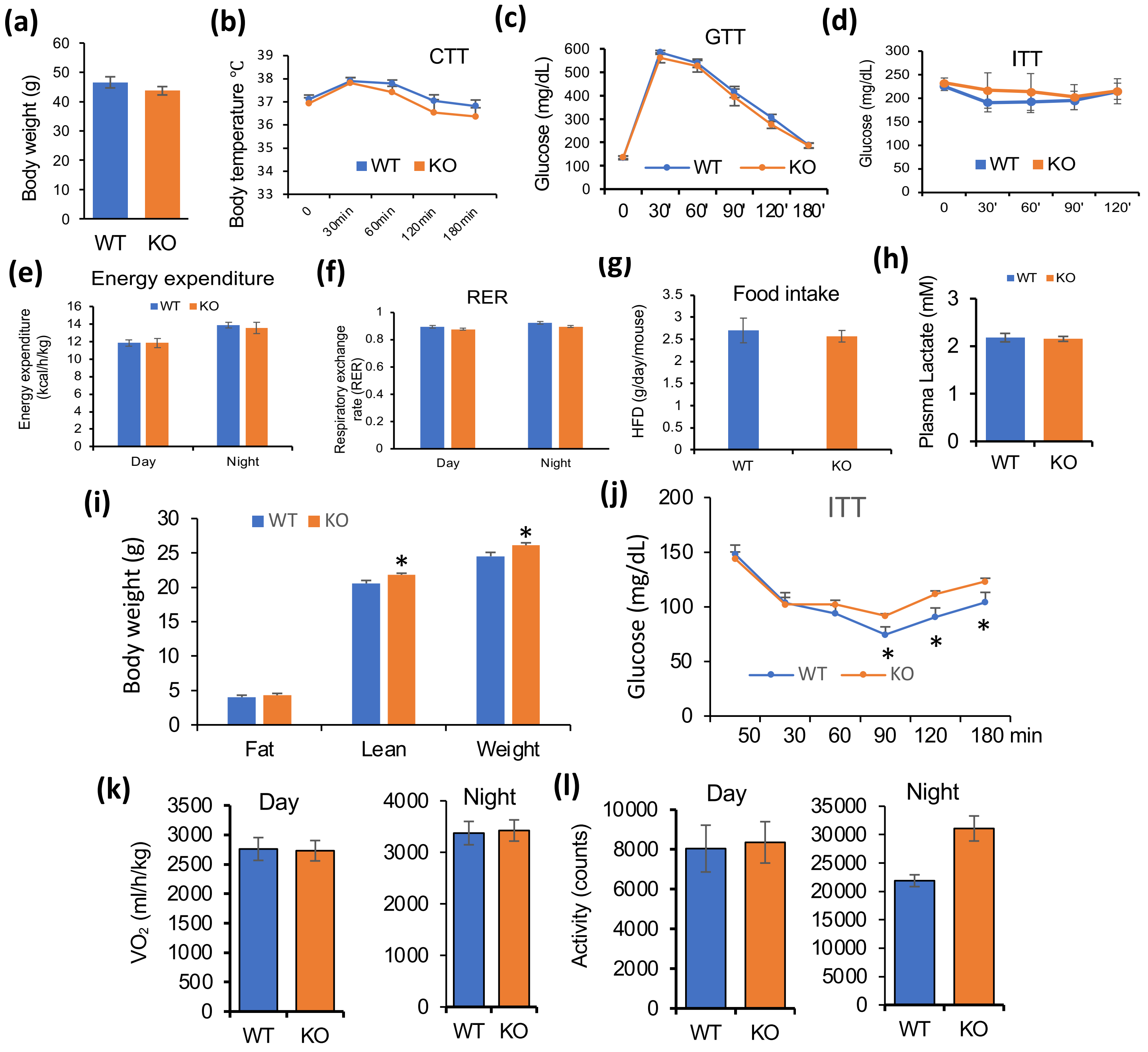

Figure S4. Metabolism in mice with adipocyte TERT KO fed HFD

AD-TERT-KO and WT littermates fed HFD for 6 months were analyzed.

(a) Body composition measured by EchoMRI in males. WT n = 7, KO n = 8.

(b) Cold tolerance test (CTT) by body temperature measured after placement at 4°C. WT n = 7, KO n = 8 males.

(c) Intraperitoneal glucose tolerance test (GTT) in males. WT n = 7, KO n = 8.

(d) Intraperitoneal insulin tolerance test (ITT) in males. WT n = 7, KO n = 8.

(e) Calorimetric measurement of energy expenditure averaged for day and night cycles (n = 3) for n = 7 WT and n = 8 KO males.

(f) Respiratory exchange ratio (RER), calculated as VCO_2/VO_2, over 3 days for n = 7 WT and n = 8 KO males.

(g) Mean daily HFD consumption over 3 days for n = 7 WT and n = 8 KO males.

(h) Circulating lactate levels in n = 7 WT and n = 8 KO males.

(i) Body composition measured by EchoMRI in WT and KO females post-HFD. n = 6.

(j) Intraperitoneal insulin tolerance test (ITT) in females post-HFD. n = 6.

(k) VO_2 in WT and KO females post-HFD during day and night cycles (n = 6).

(l) Measurement of locomotor activity in WT and KO females post-HFD (n = 6).

For all data, shown are mean+/- SEM (error bars). *$p < 0.05$ (two-sided Student's t-test).

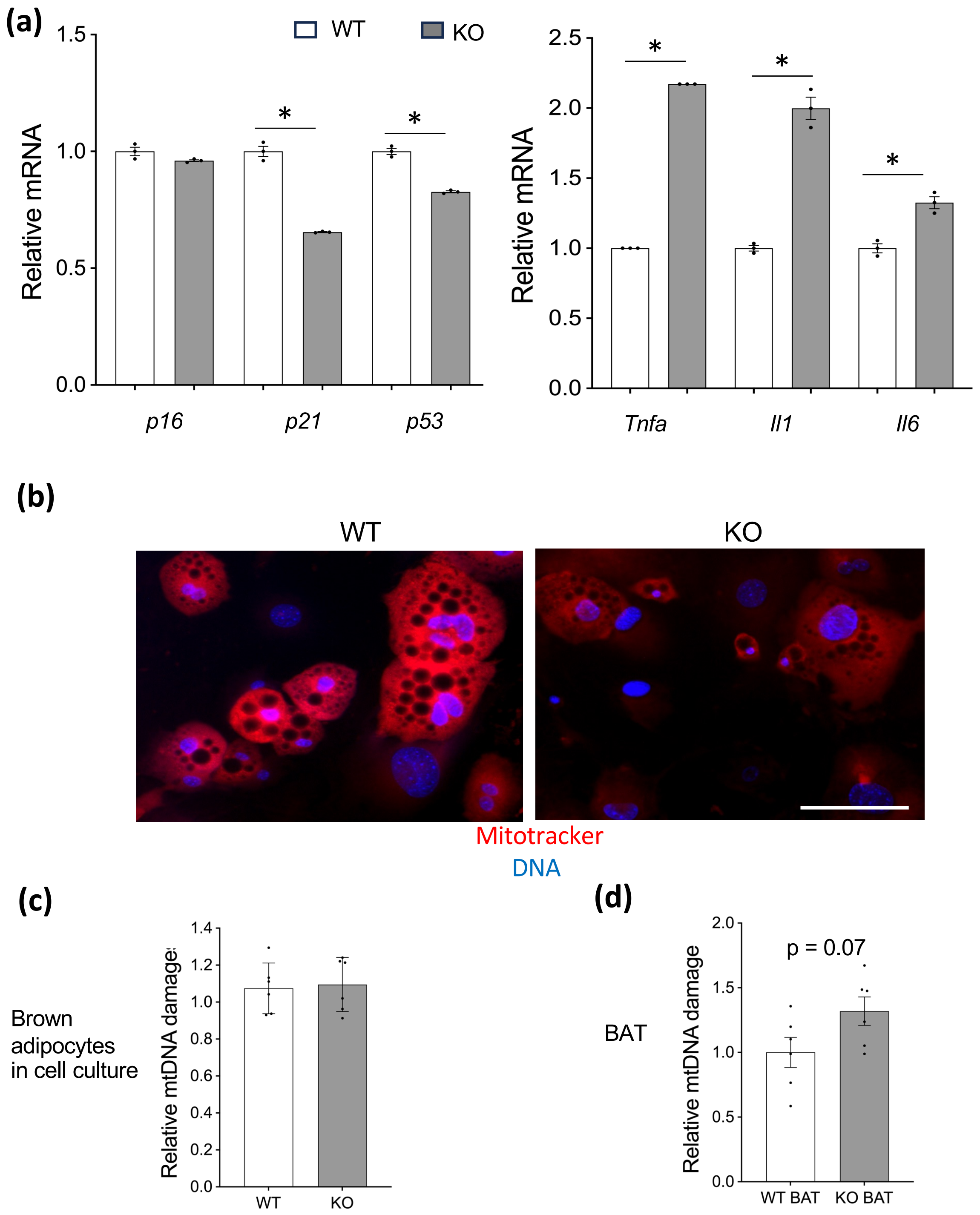

Figure S5.
TERT function in brown adipocytes. Ear fibroblasts from WT and AD-TERT-KO littermates induced for brown adipogenesis were analyzed (a-c).
(a) q-RT-PCR data (normalized to *18S* RNA) reveals a lack of senescence marker induction (left) but higher expression of SASP cytokines (right). Shown are mean+/- SEM (error bars). *$p < 0.05$ (two-sided Student's t-test).
(b) Mitotracker (red) staining reveals lower mitochondrial activity in AD-TERT-KO brown adipocytes. Scale bar: 50 µm.
(c) Mitochondrial DNA damage quantification in cultured brown adipocytes with a qPCR Assay Kit MTM-DQ-100 from RayBiotech.
(d) Mitochondrial DNA damage quantification in BAT from males fed HFD for 8 months with a qPCR Assay Kit MTM-DQ-100 from RayBiotech. n = 3 mice (duplicate PCR readings).

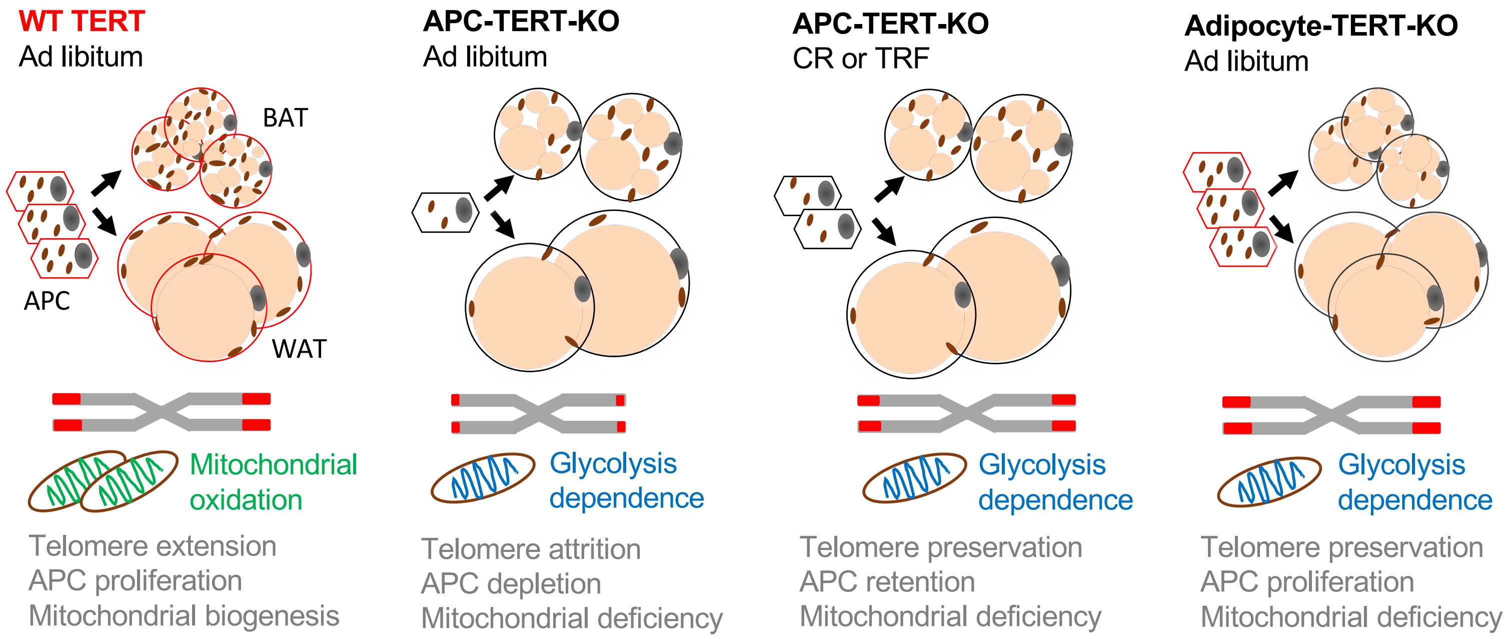

Figure S6.
A schematic of APC and Adipocyte TERT-KO models used in the study with implications on telomeres, cell numbers, and mitochondrial numbers / function in APC and adipocyte populations illustrated.
